# Shortcomings of the Commercial MALDI-TOF MS Database and Use of MLSA as an Arbiter in the Identification of *Nocardia* Species

**DOI:** 10.3389/fmicb.2016.00542

**Published:** 2016-04-21

**Authors:** Gema Carrasco, Juan de Dios Caballero, Noelia Garrido, Sylvia Valdezate, Rafael Cantón, Juan A. Sáez-Nieto

**Affiliations:** ^1^Área de Bacteriología, Centro Nacional de Microbiología, Instituto de Salud Carlos III, MadridSpain; ^2^Servicio de Microbiología, Hospital Universitario Ramón y Cajal and Instituto Ramón y Cajal de Investigación Sanitaria, MadridSpain

**Keywords:** *Nocardia* spp., MALDI-TOF MS, 16S rRNA identification, MLSA, prevalence in Spain

## Abstract

*Nocardia* species are difficult to identify, a consequence of the ever increasing number of species known and their homogeneous genetic characteristics. 16S rRNA analysis has been the gold standard for identifying these organisms, but proteomic techniques such as matrix-assisted laser desorption ionization-time of flight (MALDI-TOF MS) and housekeeping gene analysis, have also been explored. One hundred high (*n* = 25), intermediate (*n* = 20), and low (*n* = 55) prevalence (for Spain) *Nocardia* strains belonging to 30 species were identified via 16S rRNA and MALDI-TOF MS analysis. The manufacturer-provided database MALDI Biotyper library v4.0 (5.627 entries, Bruker Daltonik) was employed. In the high prevalence group (*Nocardia farcinica, N. abscessus, N. cyriacigeorgica* and *N. nova*), the 16S rRNA and MALDI-TOF MS methods provided the same identification for 76% of the strains examined. For the intermediate prevalence group (*N. brasiliensis, N. carnea, N. otitidiscaviarum* and *N. transvalensis* complex), this figure fell to 45%. In the low-prevalence group (22 species), these two methods were concordant only in six strains at the species level. Tetra-gene multi-locus sequencing analysis (MLSA) involving the concatemer *gyr*B-16S rRNA-*hsp*65-*sec*A1 was used to arbitrate between discrepant identifications (*n* = 67). Overall, the MLSA confirmed the results provided at species level by 16S rRNA analysis in 34.3% of discrepancies, and those provided by MALDI-TOF MS in 13.4%. MALDI-TOF MS could be a strong candidate for the identification of *Nocardia* species, but only if its reference spectrum database improves, especially with respect to unusual, recently described species and species included in the described *Nocardia* complexes.

## Introduction

The members of *Nocardia* are branched Gram-positive opportunistic pathogens that live in soils rich in organic matter. Nearly 90 species (Euzeby, 2013) cause clinical problems, including cutaneous respiratory and systemic disease. Over 85% of all *Nocardia* species were identified during the molecular era; previously, time-consuming phenotypic tests were used (Brown-Elliott et al., 2015). Nowadays, the sequencing of the 16S rRNA gene is the most widespread method (Brown-Elliott et al., 2015). However, other techniques based on protein profiling, such as matrix-assisted laser desorption ionization-time of flight mass spectrometry (MALDI-TOF MS), have increased their throughput and potential use (Singhal et al., 2015) and have been shown useful in the routine laboratory identification of *Nocardia* (Carbonnelle et al., 2010).

Unfortunately, the MALDI-TOF MS identification harbors limitations when only manufacturer-provided database has been applied. It was stated in a literature review displaying low values of correct *Nocardia* species assignment (≈15–53%) (Xiao et al., 2016). In addition, the constantly increasing number of, recognized *Nocardia* species means that the commercial MALDI-TOF MS spectrum database for the genus can become outdated. Both facts made that requiring laboratories to compile their own libraries to get a successful identification (Brown-Elliott et al., 2015; Buckwalter et al., 2016). However, this step could not be feasible for many of the routine clinical laboratories, in which MALDI-TOF MS has been widely introduced.

To achieve a successful species assignment in this genus, molecular techniques as full-length 16S rRNA gene or multi-locus sequence analysis (MLSA) have been undergone. Both are high cost techniques that need on-site sequencing facilities, not being available in most of the clinical laboratories (Xiao et al., 2016).

The present work compares the agreement between 16S rRNA full gene and MALDI-TOF MS identification of *Nocardia* species by using the current commercial database without in-house supplementation (MALDI Biotyper software package version 3.1) and identifies the shortcomings of the latter. This analysis has been performed in a wide population of clinical *Nocardia* strains constituted by species with different prevalence in Spain. The tetra-gene MLSA (McTaggart et al., 2010) involving the concatemer *gyrB*-16S rRNA-*hsp65*-*secA1* was used to arbitrate between discrepant identifications.

## Materials and Methods

### Bacterial Strains, Amplifications, and Sequencing

The bacteria examined in this work were 100 *Nocardia* strains recovered from clinical samples (84 of respiratory origin, 6 cutaneous, 2 from the central nervous system and 8 from other tissues) sent to our reference centre (Spanish National Centre for Microbiology) from different hospitals between 2006 and 2014. These were identified by 16S rRNA analysis (see below) as representing 30 *Nocardia* species, and were selected for the present study since they represented more and less commonly encountered members of the genus. All these bacteria were incubated on buffered charcoal yeast extract agar (BCYE) or Columbia 5% sheep blood agar at 37°C for at least 48 h (i.e., until growth was clearly visible). DNA was extracted by the boiling method and amplifications of the studied genes were performed using Ready-To-Go PCR Beads (Amersham Biosciences, Buchinghamshire, UK). The products were electrophoresed and purified using Exo SAP-ITTM reagent (GE Healthcare, NJ, USA), and sequenced by capillary electrophoresis in a ABI PRISM 3100 apparatus (Applied Biosystems, Foster City, CA, USA) (Carrasco et al., 2013) PCR primers and conditions are listed in Supplementary Table S1.

### 16S rRNA Analysis

The received *Nocardia* strains were identified at the species level by sequencing of the full-length 16S rRNA gene (size ~1215 bp) (with proofreading and editing as necessary). The obtained sequences were compared with those in the GenBank database; those showing ≥99.6% similarity were deemed to be the same and thus positively identified following CLSI MM18 guidelines (Petti et al., 2008).

**FIGURE 1:**
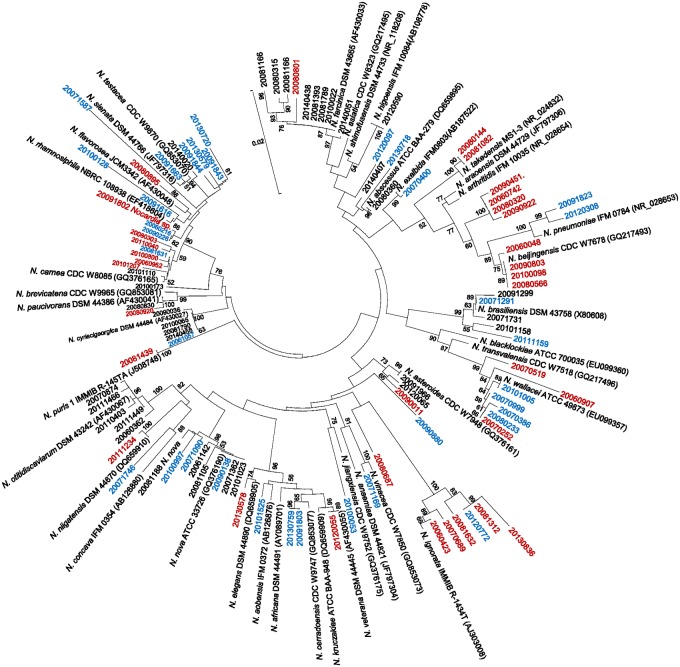
**Phylogenetic tree based on 16S rRNA analysis of 100 *Nocardia* spp. strains (collected in Spain between 2006 and 2014), plus type strains.** The reliability of the topologies was assessed by the bootstrap method with 1000 replicates. The type strains, and the species for which full agreement was reached between the 16S rRNA and MALDI-TOF MS methods, are represented in black. Strains with partially concordant identifications are shown in blue. Strains for which no agreement was reached between the 16S rRNA and MALDI-TOF MS methods are shown in red.

The BioEdit (Hall, 1999), CLUSTAL W (Thompson et al., 1994), and Mega 6.0 computer programs were used to construct a 16S rRNA phylogenetic tree (Tamura et al., 2013), employing the neighbor joining method (Gascuel, 1997) and Kimura 2 parameter distances (Kimura, 1980) (**Figure 1**). The reliability of the topologies was assessed by the bootstrap method with 1000 replicates.

Three groups were established according to the prevalence of *Nocardia* species in Spain (Carrasco et al., 2013): 25 strains representing *Nocardia abscessus*, *N. cyriacigeorgica*, *N. farcinica* and *N. nova* were assigned to the high prevalence group, 20 strains representing *N. brasiliensis*, *N. carnea*, *N. otitidiscaviarum*, and the *N. transvalensis* complex were assigned to the intermediate prevalence group, and 55 (representing 22 less frequently detected species) to the low prevalence group.

### MALDI-TOF MS Identification

Protein samples were initially prepared as previously described (Verroken et al., 2010). As seen with *Mycobacterium* spp., the analysis of *Nocardia* by MALDI-TOF MS requires somewhat complicated sample preparation (Dunne et al., 2014) due to the presence of aliphatic acids in the cell wall (Xiao et al., 2016). Despite this fact, three modifications of this extraction technique were used (see Supplementary File S1): (i) mechanical disruption with glass-beads; (ii) 10 min of sonication at 2500 oscillation min-1 using a Mini Bead Beater (Biospec Products, Bartlesville, OK) (both of these options preceding the rest of the Verroken et al. method); and (iii) freezing for 24 h before proceeding to protein extraction (performed after the boiling step described in the Verroken et al. method).

Matrix-assisted laser desorption ionization-time of flight spectra were randomly obtained from blind samples in the linear positive mode at a laser with a frequency of 20 Hz in the range of 2–20 kDa with a Microflex X instrument using the FlexControl 3.3 (Bruker Daltonik, Bremen, Germany). Each recorded spectrum is the result of six series of 40 single laser shots in different locations, with user intervention only when needed. Raw spectra of bacteria were explored using the MALDI Biotyper 3.1 software package with default settings. The database for identification was the reference Biotyper library v4.0 5.627 MSP (Bruker Daltonik). In 2014, 5627 species were included in this database (Piau et al., 2015). Identification was performed with no custom database supplementation. Identification scores (MALDI-TOF MS log scores) of ≥2.0 and 1.7 – < 2.0 indicated species-level and genus-level identifications, respectively, (Verroken et al., 2010; Hsueh et al., 2014).

### Multi-Locus Sequence Analysis; Arbitration between Discrepant Identifications

Multi-locus sequencing analysis was performed when the identifications made by the 16S rRNA and MALDI-TOF MS methods did not agree (*n* = 67). The *Nocardia* identification pattern of McTaggart et al. (2010) was used, but with four genes instead of five; 16S rRNA, *gyrB*, *hsp65*, and *secA1* genes. *rpoB* was omitted given the reliability of this four-locus technique compared to the five-locus technique (99.5%) (Liu et al., 2011). Concatenated sequences of these four genes from each discrepantly identified sample were aligned and a phylogenetic tree constructed. When type strains were not available from GenBank, those used in the McTaggart et al. study were employed (McTaggart et al., 2010). *N. elegans*, *N. takedensis*, and *N. jiangxiensis* lack a reference strain for all the genes in the MLSA pattern; they do not appear, therefore, in the phylogenetic tree. The results obtained were then compared against the discrepant identifications made by the 16S rRNA and MALDI-TOF MS methods.

### Nucleotide Sequence Accession Numbers

GenBank accession numbers for the sequences reported in this study are KT933407-KT933625.

## Results

### Identification of *Nocardia* Species via MALDI-TOF MS, Resolving Discrepancies by MLSA

**Table 1** shows the degree of overall agreement between 16S and MALDI-TOF MS identification with manufacturer-provided database (MALDI Biotyper library v4.0), and that within each prevalence group according to its frequency in Spain (Carrasco et al., 2013). The 16S rRNA method identified 79 out of 100 studied strains at the species level (Petti et al., 2008); 13 could only be identified as *Nocardia* spp. Similarity values of 99.0–99.5% were obtained for eight strains, not enough to assign them to any *Nocardia* species, although they can be assumed to belong to different *Nocardia* complexes (Schlaberg et al., 2013).

**Table 1:** Level of agreement between 16S rRNA and commercial MALDI-TOF MS database (Bruker Biotyper library v.4.0) identification of *Nocardia* species.

*Nocardia* spp. (no. of strains)	Agreement between 16S and MALDI-TOF MS	No. of strains in the Bruker database
**High prevalence (*n* = 25)**		
*Nocardia abscessus* (*n* = 2)	2/2	2
*N. cyriacigeorgica* (*n* = 6)	5/6	16
*N. farcinica* (*n* = 8)	7/8	12
*N. nova* (*n* = 9)	5/9	2
Total (%)	19/25 (76.0%)	32
**Intermediate prevalence (*n* = 20)**		
*N. brasiliensis* (*n* = 5)	3/5	1
*N. carnea* (*n* = 7)	1/7	1
*N. otitidiscaviarum* (*n* = 5)	5/5	6
*N. transvalensis* complex (*n* = 3)	0/3	1
Total (%)	9/20 (45%)	9
**Low prevalence (*n* = 55)**		
22 *Nocardia* species:	5/55	45
-13 *Nocardia* species included in Bruker database (*n* = 28)	5/23	45
-9 *Nocardia* species not included in Bruker database (*n* = 27)	0	0
Total (%)	5/55 (9.1%)	45

For the 33 strains with *concordant* 16S rRNA and MALDI-TOF MS identifications at species level, the percentage similarity of the MLSA concatemer for each strain with respect to the reference sequences in GenBank reached 93.8–98.7%, 90.7–97.4%, and 90.1–100% for the high, intermediate and low-prevalence subgroups, respectively.

For the 67 strains, *not concordant* in the identification made by the 16S rRNA and MALDI-TOF MS methods, MLSA was performed. Among them, 34 strains were *concordant* in genus but discrepantly identified at the species level by the 16S rRNA and MALDI-TOF MS. Their percentage similarities of the MLSA concatemer for each strain with respect to the reference sequences for high, intermediate and low-prevalence subgroups were of 94.4–98.9%, 96.9–97.6%, and 94.4–100.0%, respectively. MLSA confirmed 7 identifications made by 16S rRNA and 9 identifications made by MALDI-TOF MS (Supplementary Table S2).

Further 33 strains -25 from the low prevalent group- were *not concordant* even at genus level by 16S rRNA and MALDI-TOF MS. MLSA confirmed 16 identifications made by 16S rRNA and none identification was made by MALDI-TOF MS, as is shown in Supplementary Table S2. Their percentage similarities of the MLSA concatemer for each strain with respect to the reference sequences for high, intermediate and low-prevalence subgroups were of 99.8% (there was one strain only), 91.2–96.1 and 94.7–99.4%, respectively (Supplementary Table S2). *N. ignorata* was excluded from analysis given the strong presence of degenerate positions in the reference strain partial 16S rRNA gene (*N. ignorata* IMMIB R-1434T; GenBank accession no. AJ303008).

### Identification of *Nocardia* High Prevalence Group

**FIGURE 2:**
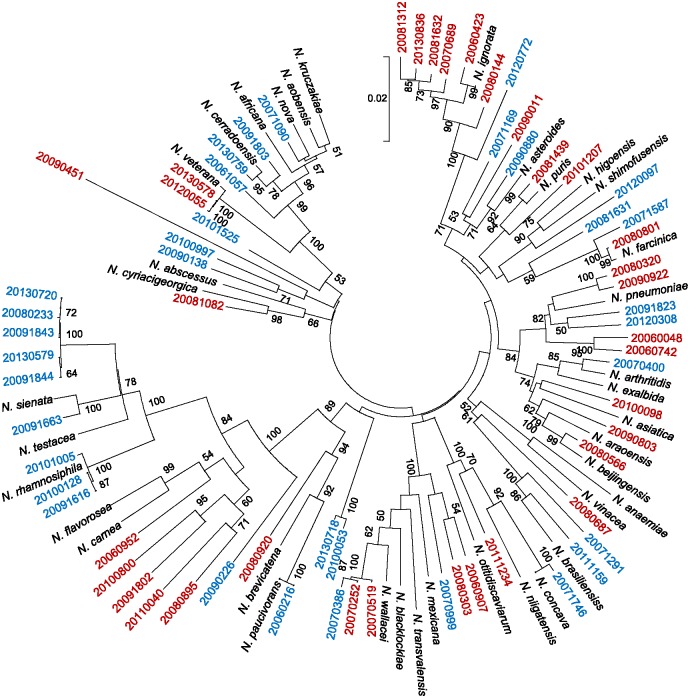
**Multi-locus sequencing analysis (MLSA)-based phylogenetic tree for 67 *Nocardia* spp. for which 16S rRNA and MALDI-TOF MS identification was discordant, plus type strains.** The reliability of the topologies was assessed by the bootstrap method with 1000 replicates. The type strains are shown in black, and strains with partially concordant identifications are shown in blue. Strains for which no agreement was reached between the 16S rRNA and MALDI-TOF MS methods are shown in red. Supplementary Table S2 contains further details.

With respect to the high prevalence group, agreement between the 16S rRNA and MALDI-TOF MS methods was reached for 19 of the 25 strains examined. However, agreement was complete only for the two strains of *N. abscessus.* One of the six discrepant strains (identified as *N. farcinica* by 16S rRNA) was not identified even at genus level by the MALDI-TOF but laid together with its respective type strain in the MLSA tree. Among the five remaining strains, only one identification was confirmed by MLSA (**Figure 2**).

### Identification of *Nocardia* Intermediate Prevalence Group

The agreement between the 16S rRNA and MALDI-TOF MS methods was reached for 9 of the 20 strains examined. However, while this agreement was complete only for the strains of *N. otitidiscaviarum*, no agreement was reached for any strain of the *N. transvalensis* complex. For the 11 strains with discrepant identification, good discrimination was reached by MLSA for *N. brasiliensis*, but not *N. transvalensis* complex. In addition, MLSA was unable to distinguish between *N. carnea* and *N. flavorosea* (**Figure 2**).

### Identification of *Nocardia* Low Prevalence Group

16S rRNA and MALDI-TOF MS analysis only agreed in the species identification of just five strains in this group: 2 out of 4 examples of *N. asteroides*, 1 out of 1 example of *N. higoensis*, 1 out of 2 of *N. paucivorans*, and 1 out of 2 *N. testacea*. For 25 of the total of 55 strains of this group, it was able to confirm their belonging to the genus *Nocardia*, but not for further 25 strains.

Only 13 of the 22 species in the low prevalence group include their mass spectra (MS) in the Bruker database v 4.0 (1–3 MS profiles per species), covering 23 out of 50 of the non-concordant strains of this group. With respect to the 13 species that were covered by the Bruker database, MLSA provided confirmation in the identifications at species level of 15 strains made by 16S rRNA analysis (MALDI-TOF MS only provided identification for 4 strains). Indeed, for *N. cerradoensis*, *N. ignorata*, and *N. pneumoniae*, MLSA provided very good discrimination. MLSA was not able to confirm the 16S rRNA identifications of *N. wallacei* or *N. blacklockiae* (MALDI-TOF MS only provided unreliable alternative identifications). MLSA tree was unable to distinguish between *N. brevicatena/N. paucivorans*, *N. arthritidis/N. exalbida*, and *N. kruckzakiae/N. aobensis/N. africana/N. nova*, as identified by 16S rRNA (again, MALDI-TOF MS only provided unreliable alternative identifications with log scores <1.7).

Among the *Nocardia* strains belonging to non-covered species by MALDI-TOF v.4.0 library (*n* = 27), MLSA tree confirmed the identity of three strains – *N. puris*, *N. rhamnosiphila*, and *N. shimofusensis* – previously identified by the 16S rRNA method, but not by MALDI-TOF MS (**Figure 2**). No spectra were available in the Bruker database for *N. beijingensis*, *N. flavorosea*, and *N. wallacei* strains either, but these did locate to the same MLSA branch as their corresponding reference sequence, thus confirming to some degree their 16S rRNA identifications. No MLSA confirmation could be made for the *N. vinacea* strains identified as such by 16S rRNA analysis or as *N. aobensis* by MALDI-TOF MS. When no pattern for a *Nocardia* sp. type strain was available, only one strain returned a MALDI-TOF MS log score of ≥2.0 allowing its identification at the species level. Two of the strains with discrepant identifications, but with MALDI-TOF MS log scores of 1.7–2.0, were identified by the latter as outside of the *Nocardia* genus. It is noteworthy that MALDI-TOF MS identified 11 *Nocardia* strains as *Lactobacillus* spp., one with a log score of 1.989 (Supplementary Table S2). However, four of the latter 11 strains also received an alternative identification as belonging to the genus *Nocardia* (log score range 1.16–1.42).

Of the 13 strains identified as belonging only to *Nocardia* sp. plus a type strain specification by the 16S rRNA method (with 99.6% similarity to their comparators in the GenBank database), although not identified as such by MALDI-TOF MS with the current commercial database, six were fully identified at the species level by the MLSA tree. Two strains with a 16S rRNA-derived similarity of 99.0 and 99.5% to *N. pneumoniae* clustered in the MLSA phylogenetic tree with the *N. pneumoniae* DSM 44730 reference strain (GenBank accession no. JF797313). Besides, one *Nocardia* sp. strain unreliably identified by MALDI-TOF MS as *N. paucivorans* – but not identified as such by 16S rRNA – clustered with *N. paucivorans* DSM 44386 (GenBank accession number AF430041) in the MLSA tree.

## Discussion

The MALDI-TOF MS method would appear to have a number of shortcomings with respect to the identification of *Nocardia* species. The Verroken et al. (2010) protein extraction protocol appears to be adequate but not for all the strains. When the unmodified method was used, 81 of the 100 strains were assigned an identification (though not necessarily correct). When those strains for which no identification was given were re-tested with either the additional glass bead, the sonication, or the 48 h freezing step, serviceable extracts were achieved for a further 10, 5, and 4 strains, respectively.

The agreement values reported by MALDI-TOF MS in an earlier study involving Gram-positive bacilli such as *Lactobacillus* spp., *Listeria monocytogenes*, and *Rhodococcus* spp. reached a much higher ≥80% (Farfour et al., 2012). However, less agreement was seen for 15 *Kocuria* (26.7%) and 74 *Nocardia* strains (14.9%) (Hsueh et al., 2014). Other authors have also indicated the inadequacy of the Bruker database for identifying *Nocardia* species, leading to identification agreements between the 16S rRNA and MALDI-TOF MS methods of just 15.6% (Segawa et al., 2015), 53.0% (Khot et al., 2015), 42.0% (Buckwalter et al., 2016), and none (Xiao et al., 2016) at the species level. However, these values were improved to 90.6, 83.1, 90.0 and 95.0%, respectively, when in-house libraries with custom spectra were included.

The present results reveal the relatively poor agreement between the two methods in terms of *Nocardia* identification at the species level. While agreement was reached for 76% of the high-prevalence group species, this figure fell to 45 and 9.1%, for those of the intermediate and low prevalence groups, respectively. The small number of MSs in the Bruker database for the high (especially for *N. nova*) and intermediate (especially for *N. carnea* and *N. transvalensis* complex) prevalence species, and for unusual and recently described species, renders the method unreliable because of the lack of identification by MALDI-TOF MS with the commercial v. 4.0 database. Moreover, in the present study, the identification of >10% of the examined strains as members of *Lactobacillus* sp., as previously reported (Hsueh et al., 2014), is of particular concern.

When identifications were discrepant in *Nocardia* species, the MLSA method was used as an arbiter, to resolve the phylogenetic resolution at the species level (Glaeser and Kämpfer, 2015). When a discrepantly identified strain clustered with a type strain in the MLSA phylogenetic tree this allowed its final identification to be made (Vasileuskaya-Schulz et al., 2011). A problem with MLSA is the inexistence of identification breakpoints when using the present concatenated sequence. In addition, there is no consensus housekeeping gene system for use with *Nocardia* species, nor have codes been assigned to alleles, nor does any sequence database exist. Even so, it is the best method available for identifying *Nocardia* species, but much more expensive and laborious than 16S rRNA or MALDI-TOF MS. It is not, therefore, routinely used in health laboratories.

## Conclusion

Until the Bruker database is amplified, the MALDI-TOF MS platforms with the Bruker Biotyper library v.4.0 cannot be considered a reliable technique as a routine method for resolving *Nocardia* sp. identification. It is specially meaningful for those unusual species -intermediate and low prevalence groups-, newly described and species included the *Nocardia* complexes. Until this occurs, health laboratories should consider retaining the use of the full-length 16S rRNA gene sequencing and the reference laboratories should confirm by MLSA this identification.

## Author Contributions

GC contributed to the study design, the acquisition, analysis, and interpretation of data, and drafting the manuscript. SV, RC, and JS contributed to the study design, the interpretation of data and revising the manuscript. JC and NG contributed to the acquisition of data. All the authors have read and approved the final draft before submission.

## Conflict of Interest Statement

The authors declare that the research was conducted in the absence of any commercial or financial relationships that could be construed as a potential conflict of interest.The reviewer MQ-B and handling Editor declared their shared affiliation, and the handling Editor states that the process nevertheless met the standards of a fair and objective review.
