# Supplementary material for: Shortcomings of the Commercial MALDI-TOF MS Database and Use of MLSA as an Arbiter in the Identification of Nocardia Species
Source: Front Microbiol. 2016 Apr 21;7:542. doi: 10.3389/fmicb.2016.00542 (PMC4838697; doi:10.3389/fmicb.2016.00542)
Supplement: Supplementary file 1 [file Table_1.DOCX]

Supplementary Material

**Shortcomings of the commercial MALDI-TOF MS database and use of MLSA as an arbiter in the identification of *Nocardia* species**

**Gema Carrasco, Juan de Dios Caballero, Noelia Garrido, Sylvia Valdezate*, Rafael Cantón, Juan A. Sáez-Nieto**

*** Correspondence:** Sylvia Valdezate: svaldezate@isciii.es

**Supplementary Table S1│** PCR primers and DNA amplification conditions for 16S rRNA partial and MLSA McTaggart *et al*. scheme.

| **Gene** | **Forward primer (5′-3′) and position** | | **Reverse primer (5′-3′) and position** | **Amplification conditions** | | | **Reference** |
| --- | --- | --- | --- | --- | --- | --- | --- |
| **16S rRNA** | Amplification: fD1 (11-30)*  AGAGTTTGATCCTGGCTCAG  Sequencing: E786F (761-778)* GATTAGATACCCTGGTAG | | Amplification: rp2 (1431-1492)*  CGGCTACCTTGTTACGACTT  Sequencing: E1115R (1075-1090)*  AGGGTTGCGCTCGTTG | 1 min 94°C; 35x 30 s 90°C, 30 s 55°C, 30 s 72°C,  1 min 72°C; | | | Drancourt *et al.*, 2000 |
|  |  |  |  |  |  |  | Baker *et al.,* 2003 |
| **Multilocus Sequence Analysis (MLSA)** | |  | | |  |  | |
| **16S rRNA** | E8F (bp 11-30)* AGAGTTTGATCCTGGCTCAG | | 534r (bp 493-509)* ATTACCGCGGCTGCTGG | 30 s 98°C; 35× 5 s 98°C,  5 s 56°C, 20 s 72°C;  1 min 72°C | | | McTaggart *et al.*, 2010 |
| ***gyrB*** | Noc-*gyrB*-F (bp 972-992)* CTTCGCCAACACCATCAACAC | | Noc-*gyrB*-R (bp 1563-1582)* TGATGATCGACTGGACCTCG | 30 s 98°C; 35× 5 s 98°C,  5 s 60°C, 20 s 72°C;  1 min 72°C | | | McTaggart *et al.*, 2010 |
|  | Noc-*gyrB*-F3 (bp 1011-1030)* CGAGGAGGGCTTCCGCGCGG | | Noc-*gyrB*-R3 (bp 1512-1532)* ATCGACTGGACCTCGTTGTTC |  |  |  |  |
| ***hsp65*** | Noc-*hsp65*-F (bp 145-164)* ACCAACGATGGTGTGTCCAT | | Noc-*hsp65*-R (bp 566-585)* CTTGTCGAACCGCATACCCT | 30 s 98°C; 35× 5 s 98°C,  5 s 54°C, 20 s 72°C;  1 min 72°C | | | McTaggart *et al.*, 2010 |
|  | Noc-*hsp65*-F2 (bp 109-129)* GTTGTCCTGGAGAAGAAGTGG | | Noc-*hsp65*-R (bp 566-585)* CTTGTCGAACCGCATACCCT |  |  |  |  |
| ***secA1*** | *secA1*-F47 (bp 413-430)* GCGACGCCGAGTGGATGG | | *secA1*-ConR2 (bp 876-896)* TTGGCCTTGATGGCGTTGTTC | 30 s 98°C; 35× 5 s 98°C,  5 s 67°C, 20 s 72°C;  1 min 72°C | | | McTaggart *et al.*, 2010 |
|  | *secA1*-F47 (bp 413-430)* GCGACGCCGAGTGGATGG | | *secA1*-ConR (bp 913-933)* GCGGACGATGTAGTCCTTGTC |  |  |  |  |

*Positions are expressed respect to *N. farcinica* IFM 10152 (GenBank accession no. NR_074702)

References:Drancourt, M., Bollet, C., Carlioz, A., Martelin, R., Gayral, J. P., and Raoult, D. (2000). 16S ribosomal DNA sequence analysis of a large collection of environmental and clinical unidentifiable bacterial isolates. J. Clin. Microbiol. 38, 3623–3630; Baker, G. C., Smith, J. J., and Cowan, D. A. (2003). Review and re-analysis of domain-specific 16S primers. J. Microbiol. Methods 55, 541–555. doi: 10.1016/j.mimet.2003.08.009
