# Supplementary material for: Shortcomings of the Commercial MALDI-TOF MS Database and Use of MLSA as an Arbiter in the Identification of Nocardia Species
Source: Front Microbiol. 2016 Apr 21;7:542. doi: 10.3389/fmicb.2016.00542 (PMC4838697; doi:10.3389/fmicb.2016.00542)
Supplement: Supplementary file 2 [file Table_2.DOCX]

Supplementary Material

**Shortcomings of the commercial MALDI-TOF MS database and use of MLSA as an arbiter in the identification of *Nocardia* species**

**Gema Carrasco, Juan de Dios Caballero, Noelia Garrido, Sylvia Valdezate*, Rafael Cantón, Juan A. Sáez-Nieto**

*** Correspondence:** Sylvia Valdezate: svaldezate@isciii.es

**Supplementary Table 2│**MLSA identification of isolates with discrepant 16S rRNA and MALDI-TOF MS identifications.

| Isolate | 16S rRNA identification  (percentage similarity with respect to the reference strain) | MALDI-TOF MS identification  (log score) | MLSA identification  (percentage similarity to corresponding GenBank multilocus sequence) | Concordance between 16S rRNA and MLSA identification | Concordance between MALDI-TOF MS and MLSA identification |
| --- | --- | --- | --- | --- | --- |
| *IDENTIFIED AT GENUS LEVEL* | | | | | |
| HIGH PREVALENCE GROUP | | | | | |
| 20061057 | *N. cyriacigeorgica* (100%) | *N. abscessus*^2^ (2.104) | *N. kruczakiae* (98.1%) | No | No |
| 20071090 | *N. nova* (99.9%) | *N. aobensis*^1^ (1.595) | *N. aobensis* (98.9%) | No | Yes |
| 20090138 | *N. nova* (100%) | *N. otitidiscaviarum*^4^ (1.971) | *N. puris/N. cerradoensis* (95.4%) | No | No |
| 20090226 | *N. nova* (100%) | *N. carnea*^1^ (1.299) | *N. testacea* (94.4%) | No | No |
| 20100997 | *N. nova* (99.4%) | *N. aobensis*^1^ (1.587) | *N. puris* (94.9%) | No | No |
| INTERMEDIATE PREVALENCE GROUP | | | | |  |
| 20071291 | *N. brasiliensis* (100%) | *Nocardia* sp. MB9090 0JTHL^2^ (1.896) | *N. brasiliensis* (96.9%) | Yes | No |
| 20081631 | *N. carnea* (99.6%) | *N. farcinica*^1^ (1.389) | *N. puris* (97.0%) | No | No |
| 20111159 | *N. brasiliensis* (99.7%) | *Nocardia* sp. MB9090 0JTHL^1^ (2.200) | *N. brasiliensis* (97.4%) | Yes | No |
| 20070999 | *N. transvalensis* complex (99.8%) | *N. africana*^1^ (1.298) | *N. wallacei* (97.6%) | No | No |
| LOW PREVALENCE GROUP | | | | |  |
| 20060216 | *Nocardia* sp. T42 (100%)* | *N. paucivorans*^1^ (1.756) | *N. paucivorans* (100%) | No | Yes |
| 20070386 | *Nocardia s*p. JCM 3016 (99.8%)* | *N. nova*^2^ (2.019) | *N. wallacei* (98.0%) | No | No |
| 20070400 | *N. exalbida* (99.9%) | *N. abscessus*^1^ (1.601) | *N. arthritidis* (98.8%) | No | No |
| 20071169 | *N. vinacea* (99.8%)* | *N. aobensis*^1^ (1.913) | *N. asteroides* (96.9%) | No | No |
| 20071587 | *Nocardia* sp. 171747 (96.7%)* | *N. farcinica*^1^ (1.746) | *N. farcinica* (99.0%) | No | Yes |
| 20071746 | *Nocardia* sp. (98.1%)* | *N. concava*^2^ (1.376) | *N. concava* (99.3%) | No | Yes |
| 20080233 | *N. wallacei* (99.8%)* | *N. sienata*^1^ (1.550) | *N. sienata* (97.0%) | No | Yes |
| 20090880 | *N. neocaledoniensis* (98.6%) | *N. asteroides*^4^ (1.309) | *N. asteroides* (98.0%) | No | Yes |
| 20091616 | *N. rhamnosiphila* (100%)* | *N. testacea*^1^ (1.501) | *N. rhamnosiphila* (100%) | Yes | No |
| 20091663 | *N. testacea* (99.8%) | *N. sienata*^1^ (1.652) | *N. sienata* (99.1%) | No | Yes |
| 20091803 | *N. cerradoensis* (99.7%) | *N. nova*^2^ (1.484) | *N. cerradoensis* (99.2%) | Yes | No |
| 20091823 | *N. pneumoniae* (99.3%) | *N. farcinica*^3^ (1.724) | *N. pneumoniae* (97.7%) | Yes | No |
| 20091843 | *N. rhamnosiphila* (99.9%)* | *N. sienata*^1^ (1.498) | *N. testacea* (97.4%) | No | No |
| 20091844 | *Nocardia* sp. FSN35 (99.6%)* | *N. testacea*^1^ (1.522) | *N. sienata* (97.0%) | No | No |
| 20100053 | *N. jiangxiensis* (100%)* | *N. elegans*^2^ (1.357) | *N. puris* (94.7%) | No | No |
| 20100128 | *N. grenatensis* (99.6%)^*^ | *Nocardia sp*^3^*.* (1.366) | *N. rhamnosiphila* (98.7%) | No | No |
| 20101005 | *N. wallacei* (99.8%)* | *N. testacea*^1^ (1.623) | *N. rhamnosiphila* (99.8%) | No | No |
| 20101525 | *N. cerradoensis* (99.5%) | *N. aobensis*^1^ (1.372) | *N. aobensis* (95.1%) | No | Yes |
| 20120097 | *N. shimofusensis* (99.8%)* | *Nocardia* sp.^2^ (1.381) | *N. higoensis* (95.7%) | No | No |
| 20120308 | *N. pneumoniae* (99.2%) | *N. aobensis*^1^ (1.647) | *N. pneumoniae* (96.5%) | Yes | No |
| 20120772 | *N. ignorata* (99.6%) | *N. asteroides*^1^ (1.382) | *N. pneumoniae* (94.4%) | No | No |
| 20130579 | *Nocardia* sp. FSN35 (99.7%)* | *N. sienata*^1^ (1.399) | *N. testacea* (97.5%) | No | No |
| 20130718 | *Nocardia* sp. 84317 (99.5%)* | *N. asiatica*^1^ (1.526) | *N. puris* (94.9%) | No | No |
| 20130720 | *Nocardia* sp*.* FSN35 (100%)* | *N*. *testacea*^1^ (1.527) | *N. testacea* (97.4%) | No | Yes |
| 20130759 | *N. cerradoensis* (99.8%) | *N. nova*^2^ (1.725) | *N. cerradoensis* (99.2%) | Yes | No |
| *MISSIDENTIFIED AT BOTH SPECIES AND GENUS LEVEL* | | | | |  |
| HIGH PREVALENCE GROUP | | | | |  |
| 20080801 | *N. farcinica* (99.9%) | *Staphylococcus capitis* ssp. *urealyticus*^2^ (1.407) | *N. farcinica* (99.8%) | Yes | No |
| INTERMEDIATE PREVALENCE GROUP | | | | |  |
| 20060952 | *N. carnea* (99.9%) | *Rhodococcus rhodochrous*^1^ (1.287) | *N. carnea* (95.7%) | Yes | No |
| 20070252 | *N. transvalensis* complex (99,7%) | *Weissella viridescens*^1^ (1,730) | *N. wallacei* (98.2%) | No | No |
| 20070519 | *N. transvalensis* complex (99,7%) | *Lactobacillus plantarum*^1^ (1,286) | *N. wallacei* (98.3%) | No | No |
| 20080303 | *N. carnea* (99.6%) | *Lactobacillus fructivorans*^1^ (1.271) | *N. wallacei* (96.4%) | No | No |
| 20100800 | *N. carnea* (99.8%) | *Lactobacillus paralimentarius*^3^ (1.428) | *N. carnea* (95.0%) | Yes | No |
| 20101207 | *N. carnea* (99.6%) | *Agromyces rhizospore*^1^ (1.360) | *N. puris* (97.2%) | No | No |
| 20110040 | *N. carnea* (99.7%) | *Lactobacillus fructivorans*^1^ (1.453) | *N. carnea* (95.6%) | Yes | No |
| LOW PREVALENCE GROUP | | | | |  |
| 20060048 | *N. beijingensis* (99.8%)* | *Lactobacillus brevis*^1^ (1.262) | *N. pneumoniae* (96.5%) | No | No |
| 20060423 | *N. ignorata* (99.9%) | *Legionella dumoffii*^1^ (1.262) | *N. asteroides* (94.2%) | No | No |
| 20060742 | *N. beijingensis* (99.7%)* | *Candida auris*^3^ (1.314) | *N. pneumoniae* (96.5%) | No | No |
| 20060907 | *N. wallacei* (99.7%)* | *Staphyloococcus haemolyticus*^1^ (1.368) | *N. wallacei* (98.2%) | Yes | No |
| 20070689 | *N. ignorata* (99.6%) | *Lactobacillus amylovorus*^4^ (1.189) | *N. ignorata* (98.9%) | Yes | No |
| 20080144 | *N. takedensis* (100%)* | *Candida krusei*^1^ (1.227) | *N. ignorata* (98.1%) | No | No |
| 20080320 | *Nocardia* sp. PK2002 (99.8%)* | *Arthrobacter stackebrandtii*^1^ (1.504) | *N. pneumoniae* (97.4%) | No | No |
| 20080566 | *N. beijingensis* (100%) | *Candida krusei*^1^ (1.421) | *N. beijingensis* (99.1%) | Yes | No |
| 20080687 | *N. asteroides* (99.3%) | *Klebsiella pneumoniae*^1^ (1.410) | *N. puris* (95.1%) | No | No |
| 20080895 | *N. flavorosea* (99.3%)* | *Kocuria* sp*.*^1^ (1.392) | *N. flavorosea* (95.6%) | Yes | No |
| 20080920 | *N. paucivorans* (99.8%) | *Arthrobacter russicus*^1^ (1.100) | *N. paucivorans* (95.7%) | Yes | No |
| 20081082 | *N. takedensis* (99.8%)* | *Candida druse*^1^ (1.277) | *N. cyriacigeorgica* (97.4%) | No | No |
| 20081312 | *N. ignorata* (99.6%) | *Trichosporon mucoides*^1^ (1.304) | *N. ignorata* (98.4%) | Yes | No |
| 20081439 | *N. puris* (99.7%)* | *Clostridium septicum*^5^ (1.376) | *N. puris* (99.2%) | Yes | No |
| 20081632 | *N. ignorata* (99.6%) | *Salmonella* sp.^1^ (1.240) | *N. ignorata* (98.9%) | Yes | No |
| 20090011 | *N. asteroides* (99.9%) | *Lactobacillus rhamnosus*^1^ (1,989) | *N. asteroides* (96.6%) | Yes | No |
| 20090451 | *Nocardia* sp. PK2002 (99.7%) | *Acidovorax temeprans*^1^ (1.343) | *N. puris* (92.3%) | No | No |
| 20090803 | *N. beijingensis* (100%)* | *Trichospora mucoides*^1^ (1.460) | *N. araoensis* (97.3%) | No | No |
| 20090922 | *Nocardia* sp. PK2002 (99.0%) | *Lactobacillus brevis*^4^ (1.459) | *N. araoensis* (96.3%) | No | No |
| 20091802 | *Nocardia* sp. T42 (99.8%) | *Lactobacillus fructivorans*^1^ (1.367) | *N. carnea* (95.3%) | No | No |
| 20100098 | *N. beijingensis* (100%)* | *Lactobacillus fructivorans*^1^ (1.228) | *N. arthritidis* (97.3%) | No | No |
| 20111234 | *N. niigatensis* (99.9%)* | *Streptomyces griseus*^1^ (1.285) | *N. niigatensis* (96.4%) | Yes | No |
| 20120055 | *N. veterana* (99.7%) | *Streptococcus mutans*^1^ (1.443) | *N. veterana* (99.4%) | Yes | No |
| 20130578 | *N. elegans* (99.7%) | *Cryptococcus uniguttulatus*^3^ (1.118) | *N. veterana* (99.2%) | No | No |
| 20130836 | *N. ignorata* (99.2%) | *Lactobacillus plantarum*^1^ (1.383) | *N. ignorata* (98.6%) | Yes | No |

*: Species not present in the MALDI-TOF MS Bruker commercial database at the time of study

^1^: Protein extraction via the Verroken *et al*. method

^2^: Protein extraction via the Verroken *et al*. method after mechanical disruption with glass beads

^3^: Protein extraction via the Verroken *et al*. method after freezing for 48 h

^4^: Protein extraction via the Verroken *et al*. method with 10 min of prior sonication
