# Supplementary material for: Shortcomings of the Commercial MALDI-TOF MS Database and Use of MLSA as an Arbiter in the Identification of Nocardia Species
Source: Front Microbiol. 2016 Apr 21;7:542. doi: 10.3389/fmicb.2016.00542 (PMC4838697; doi:10.3389/fmicb.2016.00542)
Supplement: Supplementary file 3 [file Data_Sheet_1.DOCX]

Supplementary Material

**Shortcomings of the commercial MALDI-TOF MS database and use of MLSA as an arbiter in the identification of *Nocardia* species**

**Gema Carrasco, Juan de Dios Caballero, Noelia Garrido, Sylvia Valdezate*, Rafael Cantón, Juan A. Sáez-Nieto**

*** Correspondence:** Sylvia Valdezate: [svaldezate@isciii.es](mailto:svaldezate@isciii.es)

**Supplementary file 1│** Protein extraction methods employed before the MALDI-TOF MS analysis.

**Protein extraction method 1:** Based in the Verroken *et al* protocol: *Nocardia* strains were incubated on buffered charcoal yeast extract agar (BCYE) or Columbia 5% sheep blood agar at 37°C for at least 48 h. Almost 10 colonies were scraped from the agar and added to 500 µl distilled water. They were mixed and boiled for 30 min, followed by a centrifugation at 13,000 rpm for 2 min. The supernatant was removed, and the pellet was suspended in 300 µl distilled water. Then, 900 µl of ethanol were added. Two series of centrifugation for 2 min at 13,000 rpm and complete supernatant removal lead to the dried pellet. This pellet was suspended in 50 µl of formic acid (70%) and later incubated for 15 min at room temperature. Afterward, 50 µl of acetonitrile was gently added and the mixture was centrifuged for 2 min at 13,000 rpm. The supernatant was employed for the MALDI-TOF MS analysis.
**Protein extraction method 2:** The colonies suspended in distilled water were fractured by mechanical disruption with glass-beads before 30 minutes boiling. Afterward, the rest of the Verroken *et al* method was developed.

**Protein extraction method 3:** The colonies suspended in distilled water undergone 10 min of sonication at 2500 oscillation during 1 min using a Mini Bead Beater (Biospec Products, Bartlesville, OK). After, the 30 minutes of the boiling step and the remaining Verroken *et al* method was developed.

**Protein extraction method 4:** After boiling for 30 minutes the suspended colonies, they were frozen for 24 h before proceeding to protein extraction. Then, the Verroken *et al* protocol was carried on from the removal of the supernatant.

Reference: Verroken, A., Janssens, M., Birhen, C., Bogaerts, P., Huang, T.D., Wauters, et al. (2010). Evaluation of matrix-assisted laser desorption ionization– time of flight mass spectrometry for identification of *Nocardia* species. J. Clin. Microbiol. 48: 4015–4021. doi: 10.1128/JCM.01234-10
